# Supplementary material for: The Gambia has eliminated trachoma as a public health problem: Challenges and successes
Source: PLoS Negl Trop Dis. 2022 Mar 28;16(3):e0010282. doi: 10.1371/journal.pntd.0010282 (PMC8989289; doi:10.1371/journal.pntd.0010282)
Supplement: Attachment — Submitted filename: Cover Letter - revisions.doc [file pntd.0010282.s001.doc]

Robin L. Bailey

Associate Editor

*PLOS Neglected Tropical Diseases*

Michael Marks

*Deputy Editor*

*PLOS Neglected Tropical Diseases*

November 5, 2021

Dear Dr. Bailey and Dr. Marks,

I would like to resubmit the manuscript entitled “The Gambia has eliminated trachoma as a public health problem: Challenges and successes” by Agatha Aboe, Balla Musa Joof, Sarjo Kebba Kanyi, Abba Hydara, Philip Downs, Simon Bush, and Paul Courtright based on the feedback from the reviewers.

In the revised manuscript we have made the following changes.

- Line 37 – parentheses removed
- Line 47 – corrected “Chlamydia trachomatous” to “Chlamydia trachomatis” and added “is”
- Line 48 – corrected hyphen to an em-dash
- Line 49 - changed “the clinical sign used to denote the presence of active trachoma” to “the clinical sign used for estimating the prevalence of active trachoma”
- Line 65: deleted “old”
- Lines 64-67: included the word “prevalence” to criterion (a) and criterion (b)
- Line 66: included “after having stopped MDA for at least 2 years”
- Line 68: deleted “or other”
- Line 107: changed “completion of the GET2020 exercise” to “completion of the baseline mapping exercise”
- Line 110: changed “TRABUT” to “Trabut”
- Line 119: used a lower case “a” for azithromycin
- Line 144: changed “integrated” to “included”
- Line 151: removed the inverted commas around the word surgeons
- Line 171: changed “Epilation was offered to persons with fewer than five eyelashes touching the eyeball if no eyelashes touched the cornea” to read “Epilation was offered to persons with trichiasis who did not otherwise meet the criteria for an offer of surgery”
- Line 250: modified text to reflect that surveys aren’t necessarily mandatory if other high-quality evidence can be provided
- Line 255: changed “after two years” to “after at least two years”
- Line 283: added “as a public health problem”
- Line 360: added “prevalence-based”
- Line 362: included “trachomatous”
- Line 363: changed “misalignment of survey results for TF and trichiasis” to “differential speeds at which populations reach the elimination prevalence thresholds for TF and trichiasis”.
- Challenge 7: WE have kept challenge 7 in the manuscript but have added that “It is most probable that early surveys conducted in urban areas like Banjul overestimated the prevalence of TT by virtue of their methodology and analysis plan at the time.”
- Line 386: changed “comprising of the” to “composed of”

We hope you find that these revisions address the recommendations raised by reviewers and now make the manuscript acceptable for publication.

Sincerely,

Philip Downs

Neglected Tropical Diseases, Sightsavers

23 Beverly Dr. Durham, NC 27707

Tel./Fax: 404 723-6856

Email: [philipdowns@gmail.com](mailto:philipdowns@gmail.com); [pdowns@sightsavers.org](mailto:pdowns@sightsavers.org)
